# Pilot Study of the Long-Term Effects of Radiofrequency Electromagnetic Radiation Exposure on the Mouse Brain

**DOI:** 10.3390/ijerph20043025

**Published:** 2023-02-09

**Authors:** Sonia Spandole-Dinu, Ana-Maria Catrina, Oana Cristina Voinea, Alina Andone, Speranța Radu, Cerasela Haidoiu, Octavian Călborean, Diana Mihaela Popescu, Vladimir Suhăianu, Octavian Baltag, Leontin Tuță, Georgiana Roșu

**Affiliations:** 1 “Cantacuzino” National Medical Military Institute for Research and Development, 050097 Bucharest, Romania; 2 Pathology Department, Faculty of Medicine, “Carol Davila” University of Medicine and Pharmacy, 020021 Bucharest, Romania; 3 Faculty of Medical Bioengineering, “Grigore T. Popa” University of Medicine and Pharmacy, 700115 Iasi, Romania; 4 Center of Excellence in Communications and Information Technology, Military Technical Academy “Ferdinand I”, 050141 Bucharest, Romania; 5 Department of Military Systems and Equipment, Military Technical Academy “Ferdinand I”, 050141 Bucharest, Romania

**Keywords:** radiofrequency, electromagnetic radiation, behavioral tests, mice, brain, DNA methylation

## Abstract

The increasing radiofrequency (RF) electromagnetic radiation pollution resulting from the development and use of technologies utilizing RF has sparked debate about the possible biological effects of said radiation. Of particular concern is the potential impact on the brain, due to the close proximity of communication devices to the head. The main aim of this study was to examine the effects of long-term exposure to RF on the brains of mice in a real-life scenario simulation compared to a laboratory setting. The animals were exposed continuously for 16 weeks to RF using a household Wi-Fi router and a laboratory device with a frequency of 2.45 GHz, and were compared to a sham-exposed group. Before and after exposure, the mice underwent behavioral tests (open-field test and Y-maze); at the end of the exposure period, the brain was harvested for histopathological analysis and assessment of DNA methylation levels. Long-term exposure of mice to 2.45 GHz RF radiation increased their locomotor activity, yet did not cause significant structural or morphological changes in their brains. Global DNA methylation was lower in exposed mice compared to sham mice. Further research is needed to understand the mechanisms behind these effects and to understand the potential effects of RF radiation on brain function.

## 1. Introduction

The adoption of technologies that utilize electromagnetic fields has been growing rapidly, as reflected by the number of devices that use them. The most prevalent type of electromagnetic radiation is in the radiofrequency (RF) range. As the use of these technologies has increased, there has been much debate regarding the potential biological impacts of exposure to RF radiation. One particular concern related to the effects of RF is the central nervous system, given that the use of communication devices often involves close proximity or direct contact with the head.

The brain consists of various structures, including neuronal cell bodies, dendrites and axons (which can be myelinated or not and form either sparse branches or dense fiber bundles), the extracellular brain matrix, glial cells, blood vessels, and extracellular fluid [1,2]. Each of these components has distinctive electrical parameters, performs specific functions, has particular epigenetic patterns, and expresses different genes; thus, RF may potentially impact the brain in various ways, due to these diverse cellular characteristics.

RF electromagnetic radiation has been the subject of significant research due to its potential health effects, including the possibility of causing brain modifications (reviewed in [3]). There have been reports based on animal studies suggesting that exposure to RF radiation may have behavioral effects, such as changes in memory, hyperactivity, spatial learning, locomotor activity, grooming, passive avoidance, and anxiety-like behaviors. Moreover, there is some evidence these altered behavioral patterns might be determined by structural changes in the blood–brain barrier, hippocampus, cerebral cortex, glial cells, and neurotransmitter levels in different brain regions (reviewed in [4]). However, the evidence for these effects is not yet strong enough to be considered conclusive, and therefore more research is needed to fully understand the potential effects of RF on brain function and behavior.

The epigenome represents the complete set of chemical modifications of DNA and its associated proteins and regulates gene expression. Epigenetic modulation, such as DNA methylation, represents modifications in gene expression that are not caused by changes of the underlying DNA sequence. These modifications are dynamic, can be influenced by environmental stress [5,6,7] such as pollution [8,9] and may have long-lasting effects on gene expression, playing a role in the development of various diseases [10]. RF radiation pollution is generating concern [11], yet there is very little research on the potential for RF exposure to alter DNA methylation patterns, and even less about the brain.

Most studies that examine the biological effects of RF exposure use laboratory-based exposure systems, which may differ from environmental exposure to RF radiation in real-life settings. To our knowledge, there are no studies aiming to compare the effects of RF radiation exposure generated in a real-life scenario compared to laboratory settings. Thus, the aim of this pilot study was to evaluate behavioral changes and DNA methylation changes in the brain of mice exposed to RF radiation emitted in a real-life scenario—by a Wi-Fi router instead of a laboratory device.

## 2. Materials and Methods

### 2.1. Exposure System

**Figure 1:**
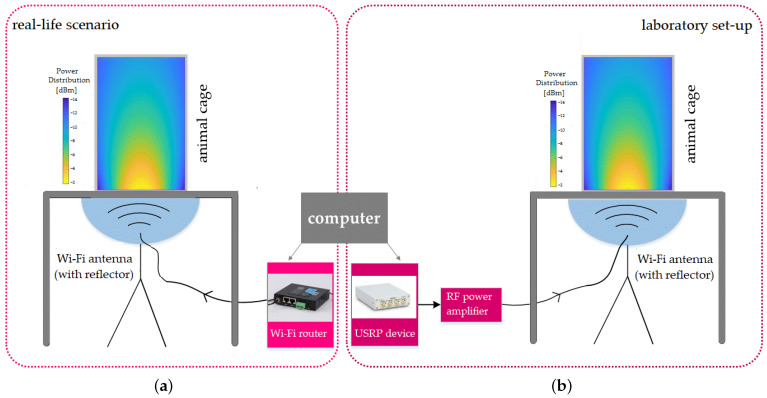
The experimental setup comprised two antenna systems each connected to a generator. (**a**) Wi-Fi router; (**b**) laboratory source.

The experimental setup aimed to accomplish two objectives: to determine the potential effects of long-term exposure to radiofrequency radiation on mice and to compare the biological responses to electromagnetic fields from real-life sources, specific to the ambient electromagnetic environment, to those generated in a laboratory setting. The exposure system, illustrated schematically in Figure 1, was composed of two 2.45 GHz whip antennas, each with a cylindrical reflector added to improve the directivity and focus the transmitted energy towards the exposed region, as well as a cylindrical enclosure holding the animals mounted above the antenna-reflector system. The 2.45 GHz frequency is used by some common wireless technologies and is typical for the IEEE 802.11 standard, which is the most commonly used standard for wireless communication.

**Figure 2:**
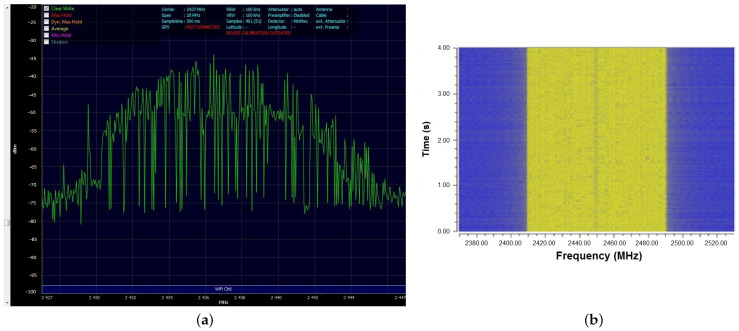
Spectrum of the signals transmitted by the two sources. (**a**) The Wi-Fi router transmission—one frequency sweep; (**b**) the laboratory source transmission variation in time (waterfall representation).

The two antennas were each connected to a radiofrequency generator: one to a USRP 2900 generator (National Instruments, Austin, TX, USA) representing the laboratory source and the other to a Wi-Fi router device (WL-520GC from Asus, Taipei, Taiwan). The Wi-Fi router was connected by cable to the internet network and wirelessly to a computer accessing a webpage streaming large data packages (such as video), in order to achieve the maximum level of transmitted power. The router used the IEEE 802.11 standard, with the following parameters: 2.4 GHz frequency band, central frequency f = 2.45 GHz, OFDM modulation, and a maximum transmitted power of 71 mW. A typical transmission signal is shown in Figure 2a. However, signals transmitted by real-world sources have high variability, as they transmit the information contained within the video data package.

The laboratory source was connected through an amplifier to the antenna and configured in the GNU Radio programming environment to transmit a frequency-modulated signal with the same parameters as the other source (the Wi-Fi router), specifically the central frequency, frequency band, and power level. The key difference compared to the real-life scenario signal is that the transmission from the laboratory source does not vary its spectrum over time, as shown in Figure 2b.

**Figure A2:**
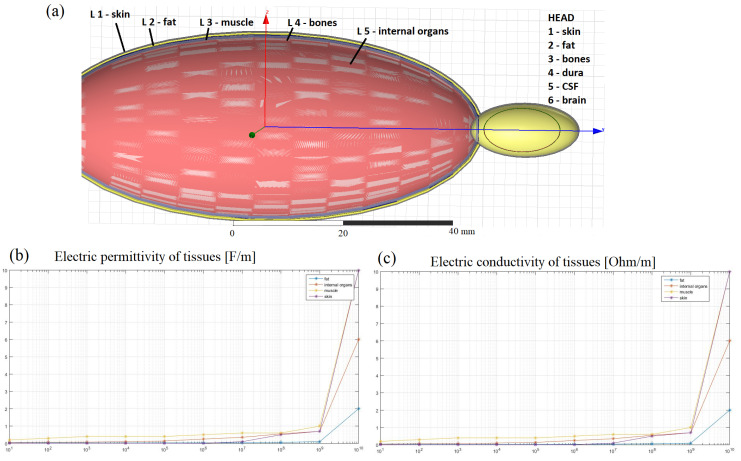
The mouse model used for SAR simulation: (**a**) the geometric model comprised of body with five layers and head with six layers; (**b**) electric permittivity of biological media depending on frequency; (**c**) electric conductivity of biological media depending on frequency.

**Figure A3:**
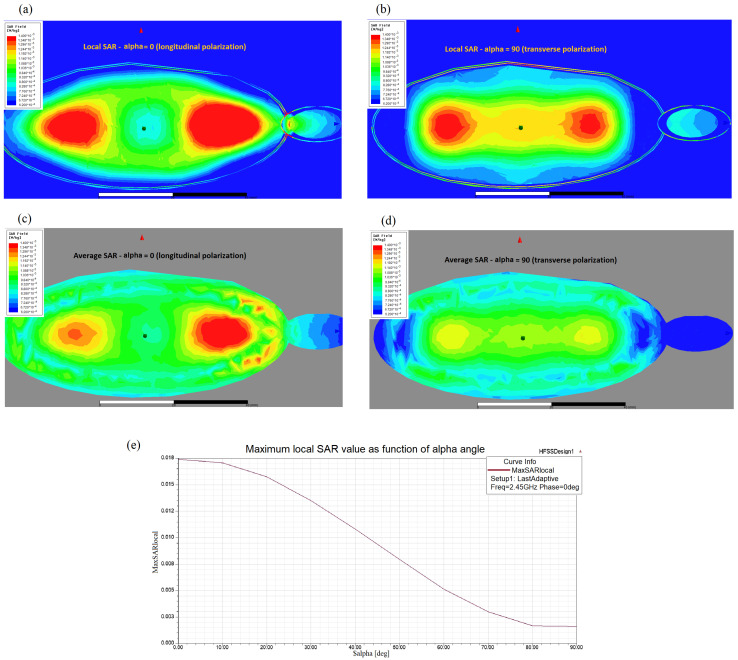
SAR distribution in the mouse’s sagittal plane: (**a**) local SAR with longitudinal polarization; (**b**) local SAR with transverse polarization; (**c**) average SAR with longitudinal polarization; (**d**) average SAR with transverse polarization; (**e**) maximum local SAR value in the sagittal plane depending on alpha angle between the field polarization and the mouse body.

The specific absorption rate (SAR) was calculated by simulating a simplified layered mouse body model (Figure A2). During the exposure, the mice were able to move freely and were randomly subjected to different field intensities and different polarizations. The worst-case scenario resulted in a maximum local SAR of 17.86 mW/kg, as shown in Figure A3. A comprehensive description of the exposure systems and SAR calculation method can be found in Appendix A.

### 2.2. Animals

This study included a total of 30 healthy male BALB/c mice, weighing between 20 and 22 g, randomly selected from the “Cantacuzino” animal facility. The mice were provided with unlimited food pellets and tap water and were kept in a controlled environment with a 12/12 h light/dark cycle, a temperature of 20–24 °C, and a relative humidity of 55–65%. After a period of adaptation lasting five days, the mice were randomly divided into a sham-exposed group (*n* = 10) and two experimental groups (*n* = 10 for each group) exposed to RF radiation emitted either by a household Wi-Fi router or a laboratory device (USRP). In order to simulate a normal, environmental exposure, mice within each group were housed together in cages and were able to move freely during the experiment. Each group was housed in a separate cylindrical glass cage with a diameter of 24 cm that was placed on top of an antenna system as depicted in Figure 1. The mice in the sham group were not exposed to RF radiation, while the mice in the RF radiation groups were continuously irradiated for 16 weeks. All experimental procedures involving animals were conducted in accordance with the European Guidelines for animal welfare (Directive 2010/63/EU) and approved by the Romanian National Sanitary Veterinary and Food Safety Authority (no. 19/12.08.2021). At the end of experimentation, all animals were humanely euthanized.

### 2.3. Behavioral Tests

All animals underwent behavioral tests at the beginning of the experiment to establish a baseline of their behavior and again at the end of the exposure period to evaluate any potential changes in behavior due to RF exposure. Locomotor activity, anxiety-related behavior, and the working memory of mice were assessed using the open-field test (OFT) and the Y-maze test. Briefly, OFT consists in placing the mice individually in one of the corners of a rectangular area (45 × 45 cm) of an open field setup (model LE800S + divider, Panlab, Barcelona, Spain) and allowing them to move freely for 10 min. The mice’s movement paths were recorded by a Monochrome CAMDCBW USB camera connected to SMART V3.0 software platform (Panlab, Barcelona, Spain) video tracker and analysis software, and the information was digitally stored. The software then determined the activity parameters such as the distance traveled and the average speed and time spent in the center and border of the OFT arena. The Y-maze test setup (model LE849, Panlab, Barcelona, Spain) was used to analyze the working memory through the assessment of spontaneous alternation behavior using the same recording and analysis setup. An alternation triplet was defined as successive entries into three different arms on overlapping triplet sets. The percentage of alternation triplets was calculated as the ratio of actual to possible alternation (defined as the total number of arm entries − 2) × 100, using the following formula: % Alternation = (Number of alternations)/(Total arm entries − 2) × 100.

### 2.4. Tissue Preparation and DNA Isolation

The mice were sacrificed at the end of the experiment, and brain tissue was harvested. For histological analysis, one hemisphere of the brain was fixed in 10% formalin and processed for paraffin embedding. The FFPE brain-tissue samples were cut into sections of 4 µm thickness using a rotary microtome (Amos Scientific, Melbourne, Australia). The sections were deparaffinized in 3 successive xylene changes of 10 min each, rehydrated in graded ethanol series down to 70%, washed in water and stained with Mayer’s hematoxylin (Bio-Optica, Milan, Italy). After treatment with lithium carbonate, the slides were differentiated with 0.5% HCl in a 70% ethanol solution and stained with an Eosin Y 1% aqueous solution (Bio-Optica, Milan, Italy). After dehydration by a series of ethanol solutions of increasing concentrations and clarification with xylene, all slides were mounted with coverslips using CV Mount (Leica Biosystems, Wetzlar, Germany) as a mounting medium. The hematoxylin-eosin (H&E) stained sections were analyzed by bright field microscopy (Zeiss LSM680) in blind by two individual researchers.

From the other hemisphere, a section spanning all brain structures weighing 20 mg was homogenized using a bead mill (SpeedMill, Analytik Jena, Thuringia, Germany) in 180 µL digestion buffer provided in the commercial kit used for DNA isolation (Invitrogen PureLink genomic DNA Mini Kit, Thermo Fisher Scientific, Waltham, MA, USA). The extraction proceeded using the manufacturer’s instructions and the DNA quantity and purity were evaluated spectrophotometrically (NanoDrop™ One, Thermo Fisher Scientific, Waltham, MA, USA).

### 2.5. Global DNA Methylation in the Brain

An enzyme-linked immunosorbent assay (ELISA) was used to measure the level of 5-methylcytosine in the brain using a commercial kit (Global DNA Methylation Assay Kit, 5 Methyl Cytosine Colorimetric, ab233486, Abcam, Cambridge, UK). To measure the methylated fraction of DNA, the DNA samples extracted from a section spanning all main brain structures were first adjusted to a concentration of 100 ng in 100 µL of binding buffer. A standard curve was also prepared according to the kit instructions. Both the standards and DNA samples were tested in duplicate. The capture and detection antibodies were used to detect the methylated DNA, which was then quantified using a colorimetric method by measuring the absorbance in a microplate spectrophotometer. The percentage of methylated DNA was proportional to the optical density (OD) measured. The OD values were converted to a 5 mC percentage by plotting them on the standard curve. The average 5 mC percentage per group was calculated and presented as the mean ± standard error of the mean (SEM).

Additionally, brain slides cut sagittally at 4 μm thickness from all animals were immunofluorescently stained for 5 mC. Briefly, sections were deparaffinized in 3 successive xylene changes of 10 min each, rehydrated in a graded ethanol series down to 70%, washed in PBS for 10 min, permeabilized with 0.1% Triton X-100 in 1× PBS (PBS-T) for 10 min at room temperature, and denatured for 30 min with freshly made 2 N hydrochloric acid (HCl) in 1× PBS in a 37 °C incubator. After denaturation, the sections were neutralized with 0.1 M Tris-HCl (pH 8.3) for 10 min, blocked with 5% normal goat serum in 0.1% PBS-T for 1 h at room temperature in a humidity chamber and then incubated overnight at 4 °C with the anti-5 mC primary monoclonal antibody (GT4111, Thermo Fisher Scientific, catalog no. MA5-31475) diluted 1:250 in blocking solution. The next day, the brain sections were washed with 0.1% PBS-T and then incubated with the secondary antibody (goat anti-mouse AF594, Thermo Fisher Scientific, catalog no. A11005) and diluted 1:500 in the blocking buffer. After the last wash, all slides were mounted with coverslips using antifade mounting medium (Leica Biosystems, Wetzlar, Germany). Slides were examined on a Zeiss LSM980 (Carl Zeiss, Jena, Germany) confocal microscope equipped with a 63×/1.4 plan-Apochromat oil differential interference contrast (DIC) objective lens using Zen Blue software. Cells in the isocortex of the mice were analyzed in order to quantify global DNA methylation. Photomicrographs were analyzed using ImageJ [12] and the 5 mC fluorescent signal was estimated by measuring the mean grey value for the regions of interest (ROI) comprising 50 cells from the isocortex of each animal. The average 5 mC per group was calculated and presented as the mean ± SEM.

### 2.6. Statistical Analysis

The statistical comparison between the three groups was analyzed using one-way ANOVA. Statistical analyses to compare the means of two groups were performed using a two-tailed *t*-test or paired sample *t*-test, as appropriate. Outliers were detected using Tukey Fence (k = 1.5) and the normality assumption was checked based on the Shapiro–Wilk test. These tests were conducted using an online calculator [13]. All data are presented as the mean ± standard error of the mean (SEM). A *p* value less than 0.05 was considered to be statistically significant.

## 3. Results

### 3.1. Behavioral Tests

#### 3.1.1. Locomotor Activity

**Figure 3:**
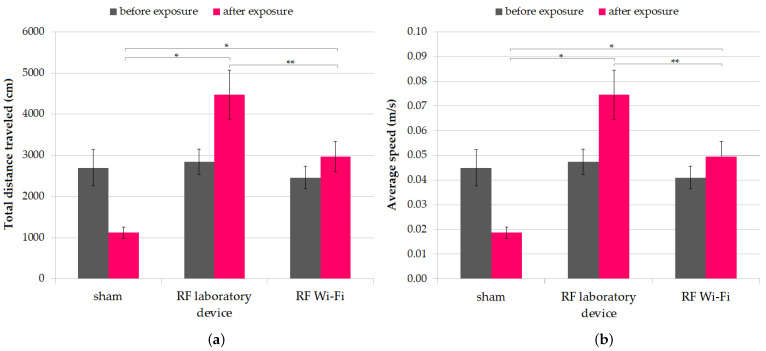
Locomotor activity of mice during the open field test (OFT). Data are represented as means and standard error of the mean (SEM). (**a**) total distance traveled measured in cm; (**b**) average speed. * *p* < 0.001, ** *p* = 0.049.

In the OFT, the three mice groups displayed similar performances at the beginning of the experiment and did not differ in terms of mean (one-way ANOVA *p* = 0.7312). However, at the end of the experiment, the locomotor activity, quantified as total distance traveled measured in cm and average speed, differed significantly between the three groups (one-way ANOVA *p* < 0.001). Analyzed individually, both means of distance traveled and average speed of mice from groups exposed to RF emitted by the laboratory device and the Wi-Fi router were significantly higher compared to sham-exposed mice (two-sample *t*-Test *p* < 0.001). Moreover, between the two RF-irradiated groups, the group exposed to the laboratory device exhibited higher locomotor activity compared to the group exposed to the Wi-Fi router (two-sample *t*-test *p* = 0.049). The results are presented in Figure 3.

#### 3.1.2. Anxiety-Related Behavior

**Figure 4:**
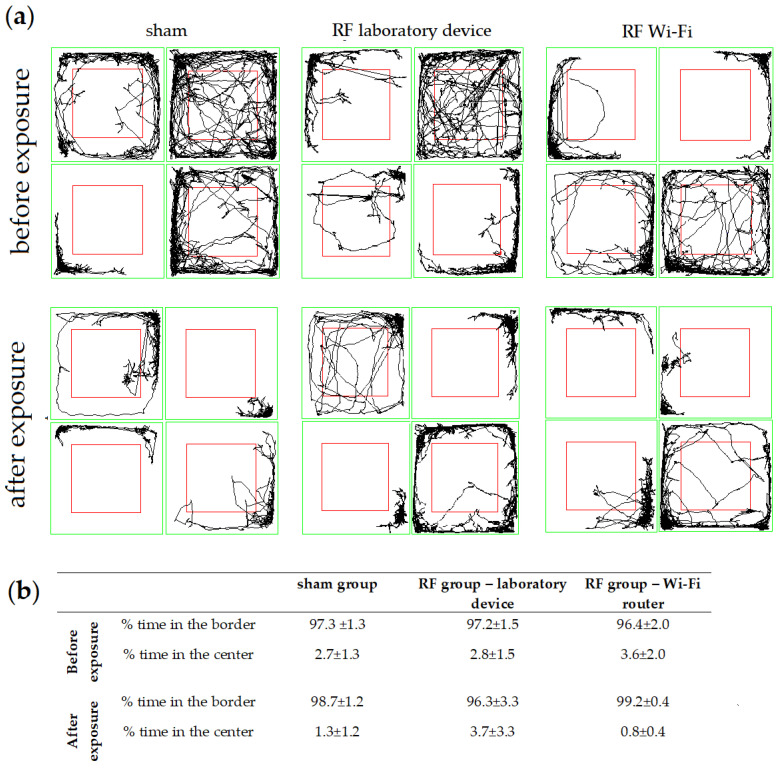
Anxiety-related behavior of mice evaluated in the OFT. (**a**) Representative trajectory diagrams of mice during the open field test (OFT); the red square delimitates the center from the border area. (**b**) Time spent in the border and center during the open field test (OFT); data are represented as mean percent and standard error of the mean (SEM).

Anxiety-related behavior was also assessed in the open-field test. In mice from all groups we observed a tendency to avoid the center of the field and to spend more time in the border. After the exposure, the percentage of time spent in the center of the OFT arena decreased slightly for mice in the Wi-Fi router RF-exposed group and in the sham group, while increasing in mice exposed to the laboratory-device-emitted RF (Figure 4). There was no significant difference in the time spent at the border vs. center between all groups, both before and after the exposure (one-way ANOVA *p* > 0.05).

#### 3.1.3. Working Memory

**Figure 5:**
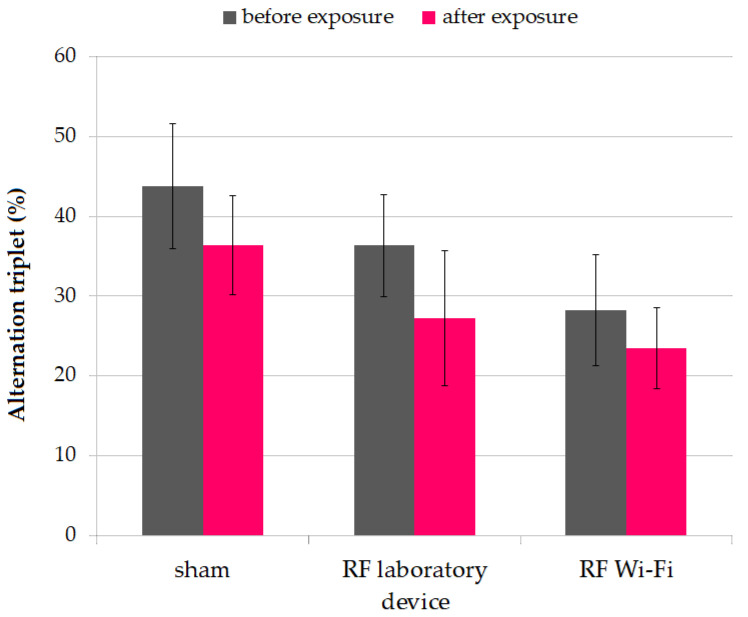
Alternation triplet percentage in mice during the Y-maze test. Data are represented as means and standard error of the mean (SEM).

We evaluated the impact of RF radiation on working memory using the Y-maze test. After the completion of the experimental period, we observed a decrease in working memory in mice from all groups, without reaching statistical significance (paired *t*-test *p* > 0.05). Additionally, neither at the beginning nor at the end of the experiment did the working memory of mice exposed to RF radiation differ significantly between groups (Figure 5).

### 3.2. Histological Evaluation

Gross morphological analysis of H&E-stained sections did not reveal any significant changes in the analyzed regions, namely the cortex, hippocampus, and cerebellum. No degenerative changes, such as expansion of the ventricles or thinning of hippocampal or cortical cell layers, were identified in any of the animals. Additionally, no signs of focal or diffuse brain injuries such as contusions, lacerations or hemorrhage were observed.

**Figure 6:**
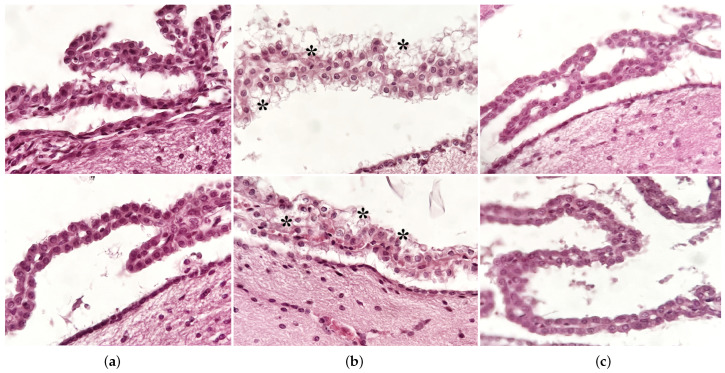
Representative micrographs of sagittal sections from mouse brain illustrating details of choroid plexuses in H&E staining. (**a**) sham group; (**b**) laboratory-device-emitted RF exposure group; (**c**) Wi-Fi router-emitted RF exposure group; * vacuolations.

Some dystrophic changes in ependymal cells, both at the level of the cerebellar cortex and especially at the level of the lateral ventricles, in the form of intracytoplasmic vacuolations, were observed in mice exposed to RF emitted by the laboratory device (Figure 6).

### 3.3. Global DNA Methylation in the Brain

**Figure 7:**
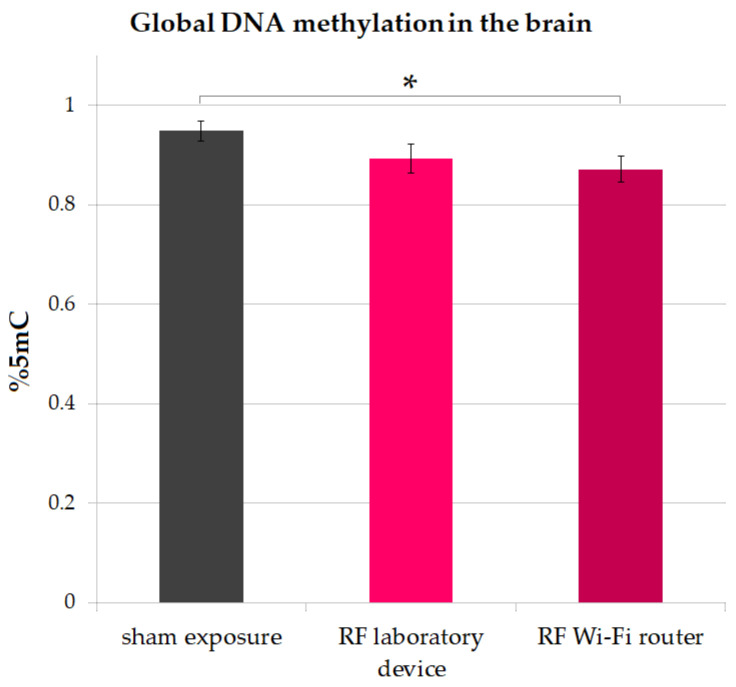
Global DNA methylation in the brains of mice, quantified as % of 5-methylcytosine (5 mC), after 16 weeks’ exposure to RF radiation. Data are represented as means and standard error of the mean (SEM). * *p* = 0.03.

Global DNA methylation was assessed at the end of the experiment by quantifying the percent of 5-methylcytosine (5 mC). Mice exposed to RF radiation emitted by the Wi-Fi router had significantly lower levels of 5 mC compared to sham-exposed mice (two-sample *t*-test *p* = 0.03). The results are illustrated in Figure 7.

**Figure 8:**
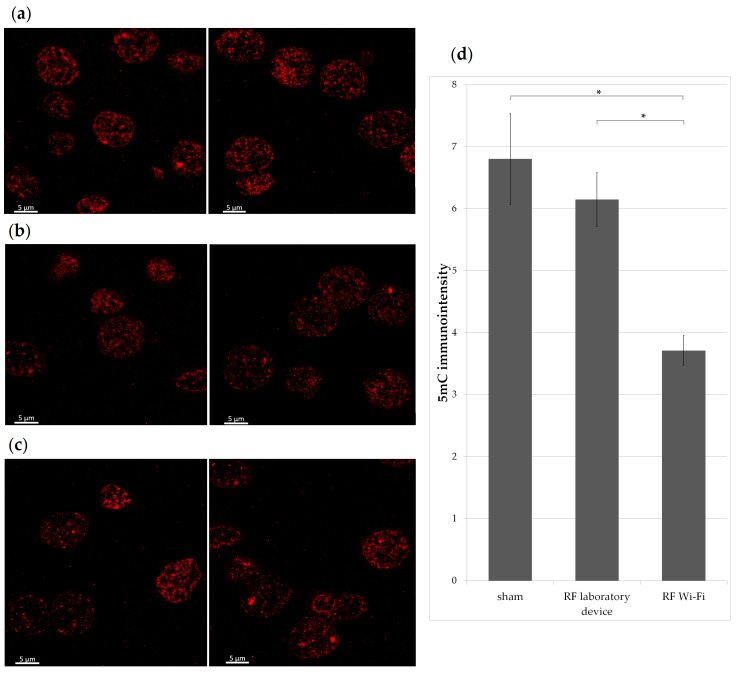
Levels of 5-methylcytosine (5 mC) in the brain of analyzed animals. Representative photomicrographs of 5 mC immunostaining in brain cross-section capturing the isocortex of mice: (**a**) sham group; (**b**) laboratory-device-emitted RF exposure group; (**c**) Wi-Fi router-emitted RF exposure group; scale bar = 5 µm; (**d**) immunointensity of 5 mC depicted by red fluorescence signals were measured and presented in a bar graph. * *p* < 0.01.

Additionally, DNA methylation of isocortex cells was quantified by immunofluorescent staining (Figure 8). There was significantly lower 5 mC in the cells of mice exposed to RF emitted by the Wi-Fi router compared to the other two experimental groups (one-way ANOVA *p* < 0.001).

## 4. Discussion

There has been increasing public concern about the potential negative effects of RF radiation on health, particularly on the brain, due to the widespread use of communication devices in daily life. To this day, the most compelling evidence correlating RF exposure to biological effects with health consequences is the presence of oxidative stress in RF-exposed animals [14,15]. Oxidative stress was associated with neurodegenerative conditions such as Parkinson’s disease, Alzheimer’s disease [16], amyotrophic lateral sclerosis [17,18], and Huntington’s disease [19]; thus, investigating further the effects of RF radiation the brain is crucial.

Concerns have also been raised about the potential behavioral effects that RF radiation exposure can have; there is evidence that exposure to GSM RF radiation can have a slight impact on attention and memory in human adults [20], yet its overall effects on behavior are debatable [21]. Additionally, in animal studies the findings are inconsistent; while some suggest RF exposure may not exert any effect on behavior [22] or have a beneficial effect by improving the behavioral impairment in animal studies [23,24,25]. As a result, the effects of RF radiation exposure on memory are still a matter of debate.

Exposure to environmental stressors and pollutants can lead to a range of structural and functional changes in the brain that are detrimental to normal functioning [26]. Electromagnetic fields have long been considered environmental pollutants [27]; thus, we hypothesized long-term exposure to RF radiation may have an impact on the behavior of exposed animals, namely mice in this study.

We exposed mice continuously for 16 weeks. This duration is considered a long-term exposure for mice considering their average lifespan and aging process [28] and covers a substantial part of a mouse’s lifespan, making it suitable for observing the long-term effects of exposure to various factors. Nevertheless, it is not of sufficient length for the mice to reach the stage of senescence, which could interfere with the interpretation of data gathered during the exposure experiment.

Our findings suggest that RF radiation exposure increased the locomotor activity of mice, as indicated by the total distance traveled and average speed during the test. To date, there are conflicting results on whether RF impacts the locomotor activity in animal models. Some studies reported no significant changes in the locomotor activity of animals exposed to RF radiation in different frequencies range (e.g., rats exposed to 1.2 GHz [29], 905-MHz [30], and 900 MHz [31], and mice exposed to 1950 MHz [32]), while others report a decrease in the locomotor activity (in rats exposed to 2.5 GHz RF radiation [33] and 900 MHz radiation [34]. There are also data suggesting RF-exposed animals have higher locomotor activity [35,36]; our results linking RF exposure with increased locomotor activity support these latter findings.

The reduction in time spent in the center of the arena in the OFT is considered a behavioral indicator of elevated anxiety levels. We did not find any significant differences between the trajectory patterns of mice from the three groups in the OFT at the beginning and end of the experiment, nor between the different groups of mice at the two time points (Figure 4).

Although we did not find a direct link between RF exposure and anxiety-like behavior in our study, we observed that mice in all groups tended to avoid the center of the arena. This suggests that there may be other factors, such as handling [37], influencing the animals’ behavior that should be taken into consideration when conducting studies and interpreting their results. There is also an ongoing debate about the extent to which the OFT accurately assesses emotionality [38]; early activity in the OFT can indicate anxiety, as it may evoke separation stress (due to the separation from cage-mates) and agoraphobia (as being exposed to a large arena that differs from the familiar holding cage) [39]. Thus, we cannot conclude whether or not exposure to RF in our experimental settings had any impact on the anxiety-like behavior of mice in our groups.

In terms of memory performance, exposure to 2450 MHz RF radiation has been found to cause spatial reference memory deficits in rats, as measured by water-maze performance [40]. Mice exposed to 800–1900 MHz cellphone signals in utero also showed impaired neurodevelopment and behavior [41]. Nevertheless, other research teams have found that exposure to 900–2450 MHz RF radiation did not cause spatial or non-spatial memory deficits in rats [42] or mice [43] when memory was evaluated through behavioral testing. Moreover, other studies reported exposure to pulse-modulated RF resulted in slower reaction times and improved accuracy in a working memory task in humans [44], while exposure to RF in adult rats has been shown to disrupt monoamine neurotransmitters, which may contribute to negative effects such as memory and learning impairments and stress [45]. Therefore, the impact of RF radiation exposure on memory remains controversial. Our findings did not reveal any significant differences in working memory in the mice at the beginning and end of the experiment, nor between the different groups of mice at the two time points.

As reviewed in recent study, locomotion in rodents is driven by complex brain-wide network; the initiation of locomotion in different higher-order states is driven by involving excitatory circuits in the cortex, midbrain, and medulla and is regulated by neuromodulatory circuits in the basal forebrain, hypothalamus, and medulla. The maintenance of locomotion involves motor, sensory, and associative cortical elements and circuits within the superior colliculus, cerebellum, periaqueductal gray, mesencephalic locomotor region, and medullary reticular formation. The ability to cease locomotion during a defensive emotional state, such as anxiety, is controlled by specific areas in the brain including the hypothalamus, amygdala, periaqueductal gray, and medullary premotor centers [46]. The neurons in the motor cortex have “memory” properties, contributing to learning or storing motor skills and behavioral adaptation [47]. Additionally, it has been shown that motor cortex of rodents, is involved in sensory guided coordination of movement, generating an appropriate behavioral response to sensory perturbations [48]. Moreover, the functional interconnections of the hippocampus with the prefrontal cortex are critically related with the integration of emotional and cognitive aspects of behavior such as working memory [49].

Here, we focused on the structural and morphological changes in the cortex, hippocampus and cerebellum. Previous studies showed long-term exposure to cellphone RF radiation at 900 MHz resulted in vacuolation diffusely in the brain parenchyma [50]. Others showed prolonged exposure to RF 900 MHz microwaves (specific absorption rate = 6 W/kg) was linked to persistent astrocyte activation in the brains of rats, which is a potential indicator of gliosis. There was no significant evidence of morphological changes or astrocyte activation in animals from this study; nonetheless, vacuolation in the ependymal cells of the choroid plexuses was observed in the animals exposed to RF emitted by the laboratory device (Figure 6), supporting previous findings [50]. The potential physiopathological effect of these changes may be related to disruptions in cerebrospinal fluid drainage, yet further testing is needed to confirm this observation and to exclude possible confounding factors.

Although there are limited data associating exposure to RF with brain structural and morphological changes, there is a line of evidence showing that exposure to RF may more commonly lead to changes in the activity of the brain (e.g., changes in electrocorticography activity of the cortex and hippocampus cells in vivo [51] and changes in the excitability of primary hippocampal neurons in vitro [52]). We did not assess the functional changes in the brains of the tested animals and further research is necessary to determine any potential connections between morphological changes and functional changes in the brain.

DNA methylation, an important epigenetic change, plays a key role in the regulation of gene expression and is involved in the development of complex behaviors [53]. Exposure to stress can alter DNA methylation patterns, potentially influencing gene expression and contributing to disease development [5,54] and behavioral changes [55,56]. The brain is composed of various types of cells, including neurons and glial cells, each of which have distinct functions and are at different stages of development. In addition, the patterns of DNA methylation vary between different types of cells [57]. In the brain, DNA methylation in the hippocampus area, more precisely in the dentate gyrus, was shown to control behavior and stress-induced gene expression [58]. DNA methylation in the neurons could be leveraged through adaptive modifications in gene expression during memory formation, and this change would be sustained as the memory is consolidated [59]. Studies have shown that cells within the isocortex [60] experience a decrease in DNA methylation as they age. Additionally, there is evidence of a naturally occurring gradient of DNA hypomethylation, particularly in excitatory neurons of mice, as they are distributed in different layers of the cortex [61].

There is limited research on the potential for RF-radiation exposure to alter DNA methylation patterns. To date, it has been suggested that RF radiation can alter DNA methylation in the estrogen receptor of colon cells of rats [62]; a single study reported modified DNA methylation patterns in the brain of rats, more precisely in the hippocampus [63] as a result of RF exposure.

Our results show a lower level of global DNA methylation quantified as the percentage of 5 mC in the brains of mice exposed to RF radiation compared to sham-exposed counterparts (Figure 7). The global levels of 5 mC in mice from the Wi-Fi router exposure group was significantly lower compared to sham (two-sample *t*-test *p* = 0.03). We found similar results when analyzing only the cells from the isocortex of mice (Figure 8), where the levels of 5 mC were notably decreased in mice exposed to the radiofrequency radiation emitted by the Wi-Fi router when compared to both a sham and laboratory-device-exposed groups (*p* < 0.001). Our results are in accordance with the previous findings reporting a hypomethylation in the hippocampus of rats exposed to RF radiation [63]; however, this change was not associated with working memory or spatial learning impairment in our groups.

One of the challenges in investigating the impact of RF radiation on behavior and the brain is that various studies have employed dissimilar devices and methods for RF radiation exposure, making it hard to match and compare the outcomes. Despite the difficulties associated with studying RF radiation, there is some evidence that it can influence the behavior of both animals and humans. To gain a more comprehensive understanding of the potential impacts of RF radiation and to increase the reliability and validity of the research, it is important for future studies to standardize as much as possible. This may include using similar devices and protocols for RF radiation exposure, as well as similar approaches when testing hypotheses.

## 5. Conclusions

Our findings showed long-term exposure to 2.45 GHz RF radiation lead to an increase in mice’s locomotor activity. These findings were not supported by changes in the morphology of the mouse brains, as no significant structural or morphological changes were observed, with the exception of some ependymal cell abnormalities. Global DNA methylation was lower in mice exposed compared to sham counterparts, supporting the previous literature. Our results indicated there are no significant differences in the effects of RF radiation emitted by the Wi-Fi router and the laboratory device. However, further research is needed to fully understand the potential effects of RF radiation on brain function.

## Figures and Tables

**Figure A1:**
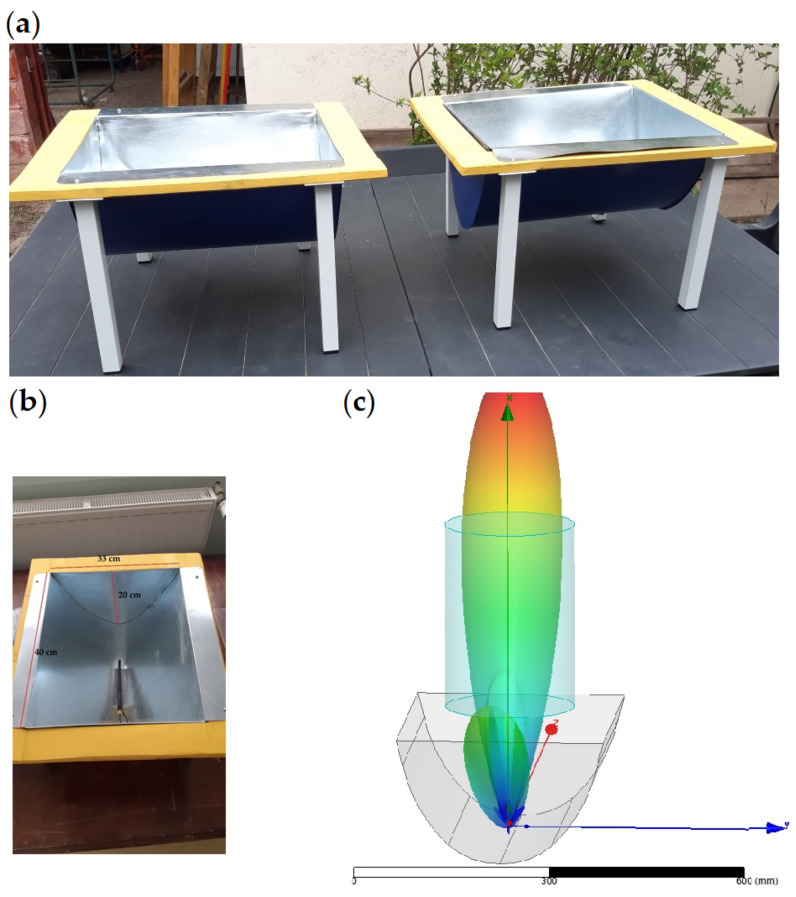
The adapted antenna system used for the experiment: (**a**) antenna systems—overall view; (**b**) dimensions of the cylindrical reflector; (**c**) simulation of the antenna system directivity with the cylindrical enclosure on top of the reflector.

## Data Availability

The data presented in this study are available on request from the corresponding author. The data are not publicly available as they contain additional information not presented and discussed in the study.

## Appendix A

The exposure system was designed for investigating the effects of long-term, continuous exposure to ambient electromagnetic fields. The standard TEM-cell setup was not suitable for this purpose, so an antenna system was adapted to reduce the stress on the animals during the experiment. The antenna system consisted of a commercial off-the-shelf Wi-Fi whip antenna and cylindrical reflectors. The whip antenna was a standard rubber duck antenna mounted on the axis of the reflector at its focus point. The parabolic cylindrical reflector had a surface contour generated according to the equation y = 0.083 × x^2^ to enhance directivity (Figure A1). The experimentally determined beam width was 2α = 25.5 degrees at the 3 dB half-power point and 2β = 46.5 degrees at 10 dB from the maximum value. The adapted antenna had a gain of G = 5.67 dBi, which is higher than the 2.5 dBi gain of a standard whip antenna.

Each antenna system used for the RF-exposure groups was connected to a generator: a standard Wi-Fi router (representing a “real-world” source) and a laboratory radiofrequency generator. The exposed animals from each group were placed in cylindrical enclosures (with 10 mice per enclosure) with a diameter of 24 cm, located about 5 cm from the reflector edge and 20 cm from the whip antenna. At this distance, the antenna beam width (2β = 46.5 degrees) was approximately 18 cm wide, covering almost the entire area explored by the mice, taking into account the thickness of the glass.

Taking into account that the spherical wave emitted by the whip antenna was reflected and transformed into a plane wave at the reflector edge, it was assumed that the far-field condition was obeyed and the Friis formula was used to calculate the power density (S) at a distance of 20 cm from the whip antenna (which was located at the center of the cylindrical enclosure), for the maximum rated power of the generator (71 mW)—the worst-case scenario. The calculated power density was S = 0.52 W/m^2^, and the corresponding electric field strength was E = 14 V/m. To calculate the specific absorption rate (SAR) in the mouse body, a simplified layered biological model was considered and constructed in the Ansys HFSS environment, which employs the frequency and time domain finite element method. The mouse body was approximated with an ellipsoidal shape that consisted of several layers, representing the skin, adipose tissue, muscles, and internal organs, as shown in Figure A2a. For the purpose of simplification, the tissues were assumed to be homogeneous, linear, and isotropic media. The tissues’ electric parameters, permittivity, and conductivity were taken from the literature [64] and their frequency dependence is depicted in Figure A2b,c, respectively.

The dosimetry analysis was focused on the worst-case scenario, using the maximum field emitted by the Wi-Fi generator. SAR was calculated for a plane wave excitation with linear polarization and electric field strength of 14 V/m, directed upwards from below the body, as in the experimental setup. The orientation of the mouse body relative to the field polarization, represented by angle alpha, varied from 0 degrees (longitudinal position, with the body parallel to the polarization) to 90 degrees (transverse position, with the body perpendicular to the polarization) as the mice were able to move freely inside the cylindrical enclosure.

Both local and average SAR was computed using different methods. The local SAR was computed for each mesh node using a simplified equation that takes into account the electric field E, the electrical conductivity σ, and the mass density ρ of the dielectric material. The average SAR was calculated using the IEEE STD P1528.4/D1.0 method. Figure A3 displays the local and average SAR in a sagittal plane for both longitudinal polarization (alpha = 0 degrees) and transverse polarization (alpha = 90 degrees). The same representation range of 0.62 mW/kg to 1.4 mW/kg was used in Figure A3 to show the higher values in the longitudinal case. To further support that longitudinal polarization represents the worst-case scenario, Figure A3e shows the maximum local SAR value in the sagittal plane as a function of the alpha angle, which varies from 0 to 90 degrees in increments of 10 degrees. The variation follows cos^2^α dependence, as described by Malus’ law of optics.

Therefore, for a single mouse exposed to the maximum power generated by the Wi-Fi router, the simplified model indicates a maximum local SAR of 17.86 mW/kg for longitudinal polarization. The mice, as they roamed freely, were exposed to various field polarizations, yet the worst-case scenario in terms of thermal effects corresponded to the electric field polarization along the mouse’s body. The maximum local SAR value obtained in the worst-case scenario was determined using an electric field strength of E = 14 V/m, which corresponds to the strongest Wi-Fi transmission at a single spectral component. For modulated transmissions, the energy is distributed across the entire channel bandwidth, resulting in a maximum power level of each spectral component in the OFDM modulated transmission that is 10 dB lower than in the previous case. As a result, the corresponding E-field peak value is approximately three times lower and the SAR is ten times lower. The laboratory source (USRP generator) was set to achieve E-field and SAR values similar to those of the average Wi-Fi transmission.
